# A Practical Treatise on Renal and Urinary Diseases

**Published:** 1872-12

**Authors:** 


### Books Reviewed.

*A Practical Treatise on Urinary and Renal Diseases, including Urinary Deposits.* Illustrated by numerous cases and engravings. By WM. ROBERTS, M. D. Second American from the Second Revised London Edition. Philadelphia: Henry C. Lea, 1872; Buffalo: T. Butler & Son.

Roberts on Urinary and Renal Diseases has been long known and prized for its completeness. The second edition has been most thoroughly revised and considerably enlarged. Two new chapters have been introduced—one on Suppression of Urine, and another on Paroxysmal Hæmatinuria. The illustrations are many of them new, and the cases reported under the different heads are interesting and instructive. The two chapters on Diabetes are full of valuable matter, and present the latest views concerning the pathology and treatment of this disease.

Chapter III. of Part II. treats in a very full and satisfactory manner on Gravel and Calculus and the medical treatment to be pursued in cases of this character. The different varieties of calculi are fully discussed, and some considerable portion of the chapter is devoted to the topic of solvent treatment of calculi.

Part III. treats of Organic Diseases of the Kidneys, and is the largest portion of the work.

Our space will not permit us to give as full notice of this work as would desire to do. It has been for some time before the public, and the profession are many of them doubtless acquainted with its merits. We can only say to those who are not in possession of a copy that the second revised and enlarged edition will afford them ample study and reflection, and present the subject of Urinary and Renal Diseases to them in its latest and most complete form.

